# Correction: Optical control of pain in vivo with a photoactive mGlu5 receptor negative allosteric modulator

**DOI:** 10.7554/eLife.34752

**Published:** 2018-01-08

**Authors:** Joan Font, Marc López-Cano, Serena Notartomaso, Pamela Scarselli, Paola Di Prieto, Roger Bresolí-Obach, Giuseppe Battaglia, Fanny Malhaire, Xavier Rovira, Juanlo Catena, Jesús Giraldo, Jean-Philippe Pin, Víctor Fernández-Dueñas, Cyril Goudet, Santi Nonell, Ferdinando Nicoletti, Amadeu Llebaria, Francisco Ciruela

Font J, López-Cano M, Notartomaso S, Scarselli P, Di Pietro P, Bresolí-Obach R, Battaglia G, Malhaire F, Rovira X, Catena J, Giraldo J, Pin JP, Fernández-Dueñas V, Goudet C, Nonell S, Nicoletti F, Llebaria A, Ciruela F. 2017. Optical control of pain in vivo with a photoactive mGlu5 receptor negative allosteric modulator. *eLife* **6**:e23545. doi: 10.7554/eLife.23545.

Published 11, April 2017

An error was identified in Figure 3D. The image showing the expression of mGlu5 receptor in primary striatal neurons mistakenly shows the expression of mGlu5 receptor in primary hippocampal neurons. The error occurred during figure assembly in PowerPoint. The accidental change in the original published figure does not affect the results and conclusions of the original paper. Thus, the aim of this Figure 3D is to illustrate that the primary striatal neurons express mGlu5 receptor.

The corrected Figure 3 is shown here:

**Figure 1:**
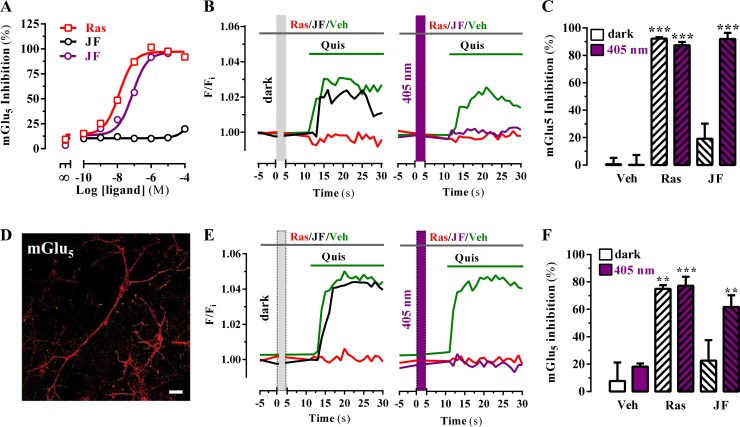


The originally published Figure 3 is also shown for reference:

**Figure 2:**
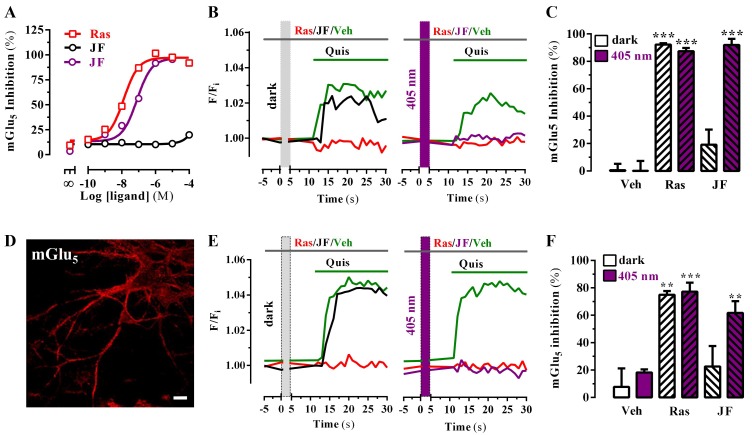


The article has been corrected accordingly

